# Case report: Backward gait training combined with gait-synchronized cerebellar transcranial alternating current stimulation in progressive supranuclear palsy

**DOI:** 10.3389/fnhum.2023.1082555

**Published:** 2023-02-22

**Authors:** Atsushi Shima, Kazuki Tanaka, Akari Ogawa, Erika Omae, Tomoaki Miyake, Yui Nagamori, Yusuke Miyata, Koji Ohata, Yumie Ono, Tatsuya Mima, Ryosuke Takahashi, Satoko Koganemaru

**Affiliations:** ^1^Department of Regenerative Systems Neuroscience, Human Brain Research Center, Kyoto University Graduate School of Medicine, Kyoto, Japan; ^2^Department of Human Health Sciences, Kyoto University Graduate School of Medicine, Kyoto, Japan; ^3^Division of Neurobiology and Physiology, Department of Neuroscience, Kyoto University Graduate School of Medicine, Kyoto, Japan; ^4^Department of Neurology, Kyoto University Graduate School of Medicine, Kyoto, Japan; ^5^Department of Electronics and Bioinformatics, Meiji University, Tokyo, Kanagawa, Japan; ^6^The Graduate School of Core Ethics and Frontier Sciences, Ritsumeikan University, Kyoto, Japan; ^7^Department of Rehabilitation and Physical Medicine, Hokkaido University Hospital, Sapporo, Japan

**Keywords:** non-invasive brain stimulation (NIBS), transcranial alternating current stimulation, cerebellum, rehabilitation, entrainment, backward gait, progressive supranuclear palsy, Parkinsonism

## Abstract

Progressive supranuclear palsy (PSP) is characterized by recurrent falls caused by postural instability, and a backward gait is considered beneficial for postural instability. Furthermore, a recent approach for rehabilitation combined with gait-oriented synchronized stimulation using non-invasive transcranial patterned stimulation could be promising for balance function. Here, we present a case of PSP with backward gait training combined with gait-synchronized transcranial alternating current stimulation (tACS). A 70-year-old woman with PSP-Richardson’s syndrome underwent backward gait training combined with synchronized cerebellar tACS. Initially, she underwent short-term intervention with combined training of backward gait with synchronized cerebellar tACS, asynchronized, or sham stimulation according to the N-of-1 study design. Synchronized tACS training demonstrated a decrease in postural instability, whereas asynchronized or sham stimulation did not. The additional long-term interventions of combined backward gait training with synchronized cerebellar tACS demonstrated further decrease in postural instability with improvements in gait speed, balance function, and fall-related self-efficacy in daily life. The present case describes a novel approach for motor symptoms in a patient with PSP. Backward gait training with synchronized cerebellar tACS may be a promising therapeutic approach.

## 1. Introduction

Progressive supranuclear palsy (PSP) is characterized by the rapid deterioration of Parkinsonism with supranuclear palsy and frontal lobe dysfunction (Höglinger et al., 2017). Postural instability is one of the symptomatic hallmarks of typical PSP with Richardson syndrome, which is closely related to quality of life (Brown et al., 2020). Although the symptoms affect activities of daily living, the effects of medication, including dopaminergic replacement therapies, are limited, and the development of training to maintain motor functions, including postural stability, is necessary.

Few studies have been conducted on rehabilitation approaches for the motor symptoms of PSP. Previous research has reported that postural reaction training with eye movements or visual awareness three times per week for 4 weeks moderately improved gait speed evaluated by 8 feet walk test (Zampieri and Di Fabio, 2008). The case report showed that 2.5-year exercise programs, including two 14 weeks of forward and backward walking training using a bodyweight-supported treadmill, maintained balance function in a patient with mixed PSP and cortico-basal degeneration (Steffen et al., 2007). Another case report showed that treadmill training for 8 weeks improved balance and gait, leading to a decrease in falls (Suteerawattananon et al., 2002). Although rehabilitation programs might be helpful in delaying the deterioration of motor symptoms, their effects seem to be limited in PSP patients (Intiso et al., 2018).

Recent rehabilitation strategies using non-invasive brain stimulation (NIBS) can be effective for gait and balance function (Forogh et al., 2017; Koganemaru et al., 2017; Krogh et al., 2022; Spiandor et al., 2022). While NIBS using anodal transcranial direct current stimulation enhances neuronal activities, NIBS using transcranial alternating current stimulation (tACS) synchronizes specific neuronal networks in a frequency and phase-dependent manner (Ali et al., 2013). The synchronization of widespread neuronal activities facilitates the transfer of information to remote areas and increases the possibility of inducing specific timing-dependent plasticity due to enhanced coincidental firing of pre- and post-synaptic neurons (Fell and Axmacher, 2011). Recently, gait-synchronized tACS over the foot area of the affected primary motor cortex (M1) improved gait and balance functions in post-stroke patients (Koganemaru et al., 2019; Kitatani et al., 2020). Cerebellar tACS has also been reported to synchronize gait cycles in healthy subjects, suggesting that the activity of gait-related neuronal networks interconnected with the cerebellar cortices was synchronized and facilitated by stimulation (Koganemaru et al., 2018, 2020).

For diseases presenting with Parkinsonism, including PSP, subclinical cerebellar involvement is reported as hypometabolism and pathological protein accumulation in cerebellar tissues (Kovacs et al., 2020) and functional impairments indicated by reduced cerebellar inhibition (CBI) (Shirota et al., 2010). The cerebellum regulates posture and balance during movements in coordination with the brainstem, which is disrupted in patients with Parkinsonism (Kurz et al., 2012; Hoogkamer et al., 2014; Peterson et al., 2014; Takakusaki, 2017). During gait, the cerebellum shows rhythmic bursts to produce step cycles and to maintain the balance of the gait-cycle-dependent swaying body in animals and possibly humans (Mori, 1987; Fukuyama et al., 1997; Mori et al., 1999). Cerebellar dysfunction leads to an inability to maintain balance during gait, resulting in a wide-based gait that enlarges the base of support in pure cerebellar dysfunction (Morton and Bastian, 2004). Compared to forward gait, backward gait is more specifically influenced by cerebellar activities that exert anticipatory postural adjustments without visual monitoring of steps and an external space (Timmann and Horak, 2001; Hoogkamer et al., 2017; Aman et al., 2018; Myers et al., 2018). Thus, backward gait training is effective in improving posture and balance, and decreasing falls (DeMark et al., 2019).

Therefore, we hypothesized that backward gait training combined with gait-synchronized tACS on the cerebellum would improve balance dysfunction, postural instability, and cerebellar activity in patients with PSP and systematically compared the short-term effects of three interventions: gait-synchronized tACS, gait-asynchronized tACS, and gait with sham tACS in a case of PSP–Richardson syndrome.

## 2. Case description

A 70-year-old woman without any medical history was referred to the hospital with difficulty in walking and recurrent falls that had deteriorated within a year. Neurological examination revealed supranuclear gaze palsy, truncal-dominant rigidity, and severe postural instability. The patient did not show any dystonia in the upper and lower extremities on the both sides. ^123^I-ioflupane single-photon emission computed tomography (Dat-SPECT) demonstrated bilateral depletion of dopamine transporters in the striatum, and MRI revealed atrophy of the midbrain tegmentum. ^123^I-IMP (N-isopropyl-p-^123^I-iodoamphetamine) SPECT revealed hypometabolism in both frontal lobes. The compound of levodopa and carbidopa was administered at a total dose of 450 mg/day, which was not effective for motor symptoms. Including levodopa-resistant motor symptoms, the patient was diagnosed with probable PSP-Richardson syndrome according to the clinical criteria (Höglinger et al., 2017). The participant was enrolled in this study a year and seven months after the onset of postural instability. The study protocol was approved by the Hokkaido University Certified Review Board of Japan (No. CRB1180001), and written informed consent was obtained from the patient (Table 1).

**TABLE 1:** Clinical course of the case.

Years	Clinical findings
0 (onset)	Aware of difficulty in walking
0.6	Developed the postural instability and recurrent falls especially to the backward
0.8	Referred to the outpatient clinic of Kyoto University Hospital Neurological findings: ● Vertical gaze palsy with preserved oculocephalic reflex ● Severe rigidity of the neck ● Retropulsion ● Dysarthria MRI: ● Atrophy of the bilateral frontal lobe, and midbrain tegmentum ^123^I-IMP SPECT: ● Hypometabolism of the bilateral frontal lobes ^123^I-ioflupane SPECT (Dat-SPECT): ● Bilateral depletion of dopamine transporters in the striatum Diagnosed as PSP with Richardson syndrome
1.0	Administration of the levodopa
1.6	Enrolled the study PSPRS was rated as 33

## 3. Diagnostic assessment

### 3.1. Clinical measurements

Clinical evaluations were performed before and after intervention. For the short-term evaluations, assessments included the timed up and go test (TUG), the mini-Balance Evaluation Systems Test (mini-BESTest) (Horak et al., 2009), and the Visual Analog Scale (VAS; score was determined by the distance on the 10 cm line, in which “0” indicated the worst condition and “10” indicated the best condition for the subjective judgment to the general motor function). For the long-term follow-ups, the Progressive Supranuclear Palsy Rating Scale (PSPRS) (Golbe and Ohman-Strickland, 2007), Fall Efficacy Scale (FES) (Tinetti et al., 1990), modified FES (Hill et al., 1996; Delbaere et al., 2011), and Activities-Specific Balance Confidence (ABC) Scale (Powell and Myers, 1995) were assessed. All the clinical evaluations were double-blinded.

### 3.2. Cerebellar brain inhibition (CBI)

To assess right cerebellar function, CBI was measured using the paired transcranial magnetic stimulation (TMS) technique (Magstim^®^ BiStim^2^, Magstim Co. Ltd., UK) (Ugawa et al., 1994) for long-term evaluation. TMS pulse on the left primary motor cortex with intensity to evoke ∼1.0 mV peak-to-peak amplitude (SI 1 mV) which was preceded by the stimulation on the right cerebellar hemisphere at 90% intensity of resting motor threshold with an inter-stimulus interval (ISI) varying between 3, 5, and 10 ms were applied for 15 times for each ISIs. The figure-eight coils were placed on the primary motor cortex and cerebellum (Benussi et al., 2017). Motor evoked potentials were recorded using electrodes placed on the right first dorsal interosseous (FDI) muscle.

### 3.3. Intervention with tACS procedure

tACS was administered using a DC stimulator (NeuroConn DC, GmbH, Germany), according to a previously described gait-synchronized stimulation (Koganemaru et al., 2019). The stimulus electrode was placed on the right cerebellum (5 × 5 cm), which was determined with a neuro-navigation system using her head MRI scan (Brainsight Brainbox Ltd., UK). We chose the right side due to more severe motor symptoms on the right side compared with the left side. A reference electrode (7 × 5 cm) was placed on the left shoulder.

The current waveform was a sinusoidal wave with a peak-to-peak amplitude of 3 mA (−1.5 ∼ +1.5 mA) computed with an external computer, and the waveform was applied to the DC stimulator. Prior to the first intervention, we determined the stimulus frequency of the tACS by calculating the gait frequency (Hz) [= 1/one gait cycle time (s)] during a 30-s backward walking at a self-speed. After that, we gave the tACS with the determined frequency during a 2-min backward walking to detect the phase of tACS to synchronize the timing of the gait cycle of the right leg (the timing of the right heel contact) using a flat pressure sensor attached to the right heel (MF01-N-221-A01, Switch Science Inc., Japan). This was because the synchronized phase of cerebellar tACS was individually different, according to a previous study (Koganemaru et al., 2020). For the calculation of the synchronized phase, all instantaneous phases of the tACS at every right heel contact were determined and summarized as a histogram with 18 discrete bins (20^°^ each). The mean phase of the bin representing the maximum number of right heel contacts was regarded as the synchronized phase.

First, we evaluated the effects of short-term intervention of backward gait training combined with cerebellar tACS according to the N-of-1 study design (Guyatt et al., 1990). We performed a combination of sham stimulation (Intervention A), gait-synchronized cerebellar tACS (Intervention B), and cerebellar tACS asynchronized with gait using the inverted phase as a control condition (Intervention C). The order of the interventions was A–C, with an interval of more than a week between the interventions. One session of backward training comprised a 4-min self-paced backward gait on the treadmill using *Kineassist* with truncal belts (Woodway USA, Inc.) to prevent falls and a 1-min break, and four sessions were performed in each intervention. In addition, the long-term intervention was performed with 10 times of the backward gait training combined with gait-synchronized cerebellar tACS twice a week for 5 weeks. The medication with a compound of levodopa and carbidopa (150 mg, three times a day) was continued during the interventions.

## 4. Results

The patient did not report any perception during all the stimulation conditions in the short-term and long-term interventions. There was neither side effect nor unanticipated event during all the interventions.

### 4.1. Results for the short-term intervention

**FIGURE 1:**
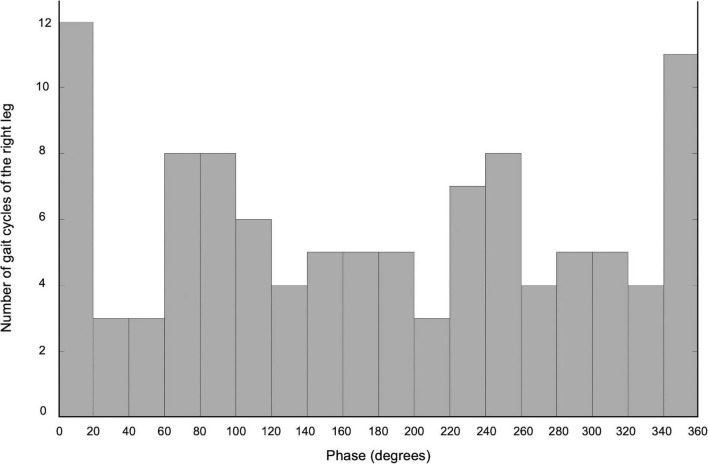
The histogram of the number of gait cycles of the right leg during the tACS X-axis demonstrates the phase of the cerebellar tACS, and Y-axis shows the number of gait cycles of the right leg.

The self-paced speed of the backward gait on the treadmill was 0.3 m/sec and the stimulus frequency of the tACS was 0.97 Hz. The phase of cerebellar tACS to synchronize the gait cycle of the right leg was 10° (Figure 1), which was used in Intervention B. The inverted phase (190°) was used as the control condition in Intervention C.

**FIGURE 2:**
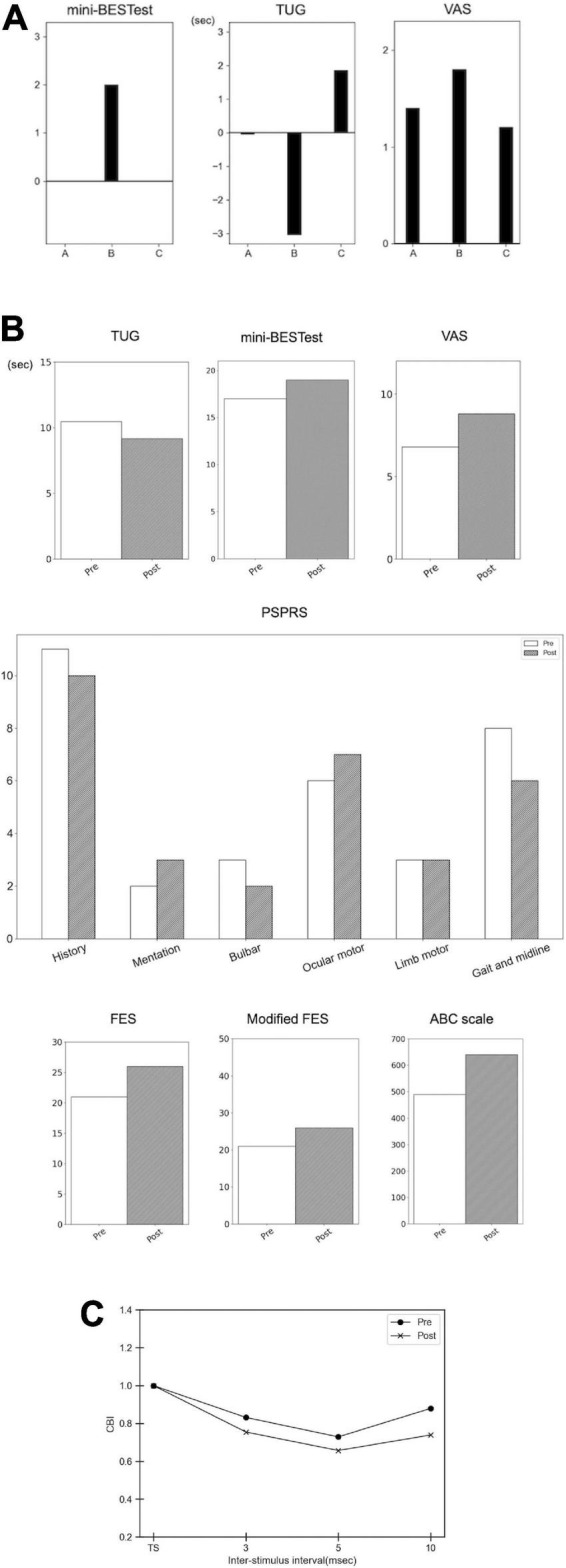
Results of the short-term intervention, long-term intervention, and cerebellar brain inhibition. **(A)** Results of the short-term intervention. The bar graph demonstrates the difference in the pre- and post-intervention (subtraction of the scores, i.e., post-minus pre-interventions). Intervention A, sham stimulation; intervention B, gait-synchronized cerebellar tACS; and intervention C, gait-asynchronized cerebellar tACS. **(B)** Results of the long-term intervention. The bar graph demonstrates the scores of mini-BESTest, VAS, the time for TUG (seconds), the sub-scores of Progressive Supranuclear Palsy Rating Scale (PSPRS), Fall Efficacy Scale (FES), modified FES, and Activities-Specific Balance Confidence (ABC) Scale in the pre- and post-interventions. **(C)** Cerebellar brain inhibition (CBI). The X-axis shows the inter-stimulus intervals, and the Y-axis demonstrates the CBI. TS, test stimulus.

The short-term intervention elucidated that Intervention B improved the time of TUG and the total score of the mini-BES test, whereas interventions A and C did not (Table 2 and Figure 2A, the baselines of TUG:10.91, 13.84 and 8.82 s, mini-BESTest:17, 15, and 17 in Interventions A–C, respectively. The VAS revealed the largest improvement in general motor symptoms in Intervention B (Figure 2A; baseline points were 7.6, 8.2, and 8.8 in Interventions A–C, respectively).

**TABLE 2:** Sub-scores of mini-BESTest.

	Items	1	2	3	4	5	6	7	8	9	10	11	12	13	14
**Short-term intervention**															
A	Pre-intervention	2	1	1	0	0	1	2	1	2	2	2	1	2	0
	Post-intervention	2	0	1	1	0	0	2	2	2	2	2	1	2	0
B	Pre-intervention	2	0	0	0	0	0	2	2	2	2	2	1	2	0
	Post-intervention	2	0	1	1	0	0	2	2	2	2	2	1	2	0
C	Pre-intervention	2	0	1	1	1	0	2	1	2	2	2	1	2	0
	Post-intervention	2	0	1	1	1	0	2	1	2	2	2	1	2	0
**Long-term intervention**															
	Pre-intervention	2	0	0	1	1	0	2	2	2	2	2	1	2	0
	Post-intervention	2	0	1	1	1	1	2	2	2	2	2	1	2	0

Items: 1. Sit to stand, 2. Rise to toes, 3. Stand on one leg, 4. Compensatory stepping correction-forward, 5. Compensatory stepping correction-backward, 6. Compensatory stepping correction-lateral, 7. Stance (feet together): eyes open, firm surface, 8. Stance (feet together): eyes open, foam surface, 9. Incline-eyes closed, 10. Change in gait speed, 11. Walk with head turns-horizontal, 12. Walk with pivot turns, 13. Step Over Obstacles, and 14. Timed up and go with dual task (3-meter walk).

### 4.2. Results for the additional long-term intervention

In addition, we evaluated the long-term intervention of backward gait training combined with gait-synchronized tACS in an observational study. We found improvements in gait and balance functions evaluated using TUG and mini-BESTest (Table 2 and Figure 2B). PSPRS was also improved, especially in the subscale of the “Gait and midline,” along with the VAS improvement for general symptoms (Figure 2B). The FES and modified FES scores increased after long-term intervention. The ABC scale also showed an increase in scores: 490 at pre-intervention and 640 at post-intervention (Figure 2B). CBI using paired TMS of the left M1 and right cerebellum was improved at inter-stimulus interval of 3,5, and 10 ms, suggesting that the function of the right cerebellum was recovered (Figure 2C).

## 5. Discussion

The present case demonstrates the potential therapeutic effect of backward gait training with synchronized cerebellar tACS in a patient with PSP. The short-term evaluation showed that training with the synchronized tACS seemed effective in improving the balance functions evaluated by TUG and the mini-BESTest, whereas sham- or asynchronized- tACS combined with backward gait did not improve them. The long-term intervention of synchronized tACS combined with backward gait training also improved gait speed and postural instability, as evaluated by TUG and mini-BESTest, and the general motor symptoms of PSP evaluated by PSPRS. The self-efficacy related to fall prevention showed an improvement in the scores for FES, modified FES, and ABC scales. Cerebellar function evaluated by the CBI was improved on the stimulated right side.

There are several approaches for the application of NIBS to the cerebellum in patients with PSP. However, their therapeutic effects on motor symptoms remain limited. One session of intermittent theta burst TMS (iTBS) on the cerebellum improved postural instability, but its long-term effects were not investigated (Pilotto et al., 2021). In an observational study, iTBS on the cerebellum improved only dysarthria, as evaluated by the PSPRS (Brusa et al., 2014).

NIBS can enhance motor function recovery in combination with rehabilitation programs (Koganemaru et al., 2015). Therefore, it would be appropriate to combine motor training with specific functions. Backward gait training has been reported to improve balance function as well as gait capacity in post-stroke patients (Wen and Wang, 2022), and to improve the stride length reflecting the gait-related balance function, compared with forward gait training in patients with Parkinson’s disease (Grobbelaar et al., 2017). Therefore, the combination of tACS of the cerebellum with backward gait training may have improved cerebellar motor control related to balance stability, resulting in improved axial symptoms of PSP in this case. Previous reports have demonstrated cerebellar dysfunction by reduced CBI in PSP patients (Shirota et al., 2010; Benussi et al., 2019). Cerebellar iTBS to induce LTP effects improved CBI without any improvement in balance function (Brusa et al., 2014). In the present case, CBI was improved at the stimulated side after long-term intervention. This suggests that improvement of cerebellar function may correlate with motor improvement due to cerebellar stimulation combined with specific training requiring cerebellar control.

The self-efficacy scores related to preventing falls showed an increase, as represented in the scores for the FES, modified FES, and ABC scales. The increase in scores may reflect a reduced risk of falls in daily living. The frequency of falls remained at zero during the interventions. The increased subjective confidence in preventing falls might have been related to the prevention of falls.

The short-term evaluation with asynchronized-tACS with the inverted phase demonstrated the deterioration of TUG. A previous report showed that asynchronized tACS over the regions between fronto-parietal areas with the inverted phase deteriorated the cognitive performance by decoupling relevant brain rhythm (Polanía et al., 2012). Similarly, it may be possible that the cerebellar function was temporally exacerbated due to decoupling the cerebellar activity with the gait rhythm to deteriorate the gait and balance function in the TUG test. Detailed assessments of cerebellar function are required in the stimulation with the inverted phase during the gait.

We applied the measurement of the mini-BESTest. The mini-BESTest is considered sensitive in the assessment of Parkinson’s disease to discriminate between fallers and non-fallers (Leddy et al., 2011), suggesting its high sensitivity of balance dysfunction in patients with Parkinsonism. Although some items (e.g., lateral push and release, standing on foam with eyes closed) are hard to perform in PSP-Richardson syndrome (Dale et al., 2022), this patient could complete almost all the assessments. The “reactive postural control” sub-scores of the mini-BESTest showed improvements only in the intervention B (real stimulation) of the short-term intervention. Therefore, we considered that the mini-BESTest could appropriately evaluate for the improvement of the balance function in this case.

The long-term intervention was performed as an additional observational study. A previous observational study and case reports showed an improvement of the balance function or fall tendency in PSP patients by the long-term exercise program (Matsuda et al., 2022) or gait training (Suteerawattananon et al., 2002; Steffen et al., 2007). Therefore, the practice effect may affect the performance after the intervention in this case. A comparative study is warranted by conducting long-term backward gait trainings with or without the gait-synchronized brain stimulation.

The spatial resolution of the tACS is limited (Yang et al., 2021) and the scalp-applied currents are attenuated to 25% by soft tissue and the skull (Miranda et al., 2006; Wagner et al., 2007; Vöröslakos et al., 2018). However, we confirmed the peak intensity of the electric field on the right cerebellum and less distribution on other brain areas by simulating the electric field distribution in the patient’s head MRI with the current electrode montage (Saturnino et al., 2019; Puonti et al., 2020).

As typical PSP cases demonstrate mild atrophy of the brainstem and cerebellum (Mimuro and Yoshida, 2020), this case showed mild cerebellar atrophy. Meanwhile, the peak of electrical field was simulated on the right cerebellum and the cerebellar-brain inhibition was enhanced after the long-term intervention. Therefore, we considered that the application of tACS with the current electrode montage could modify the cerebellar activity.

In normal gait, the cerebellum plays a pivotal role in predictive feedforward adaptation to novel terrains and environments (Takakusaki, 2013; Pisotta and Molinari, 2014). A previous study showed that patients with cerebellar impairment had a difficulty or inability in feedforward prediction and adaptation relating to the lower-limb and posture controls during the gait on the split-belt treadmill (Morton and Bastian, 2006). Compared to forward gait, backward gait needs more precise feedforward postural and lower-limb adaptation by the cerebellum due to the controlling the body and limbs without visual information (Klous et al., 2011). Then, the present intervention with cerebellar tACS and backward gait training may have contributed to improvements of the predictive feedforward adaptation by the cerebellum.

The present case demonstrates that backward gait training combined with synchronized cerebellar tACS can be a promising treatment for improving the motor symptoms of PSP.

## Author’s note

The patient stated that she hoped that the present findings may contribute to the development of new strategies for the treatment of PSP.

## Data availability statement

The raw data supporting the conclusions of this article will be made available by the authors, without undue reservation.

## Ethics statement

The studies involving human participants were reviewed and approved by Hokkaido University Certified Review Board of Japan. The patient provided her written informed consent to participate in this study. Written informed consent was obtained from her for the publication of any potentially identifiable images or data included in this article.

## Author contributions

AS and SK designed the study, collected and interpreted data, and contributed to the initial draft of the manuscript. AS, AO, TMiy, YN, YM, KO, and YO contributed to data collection and analysis. TMim and RT contributed to the interpretation and critically reviewed the manuscript. All authors approved the final version of the manuscript.
